# Food Sensing: Detection of *Bacillus cereus* Spores in Dairy Products

**DOI:** 10.3390/bios10030015

**Published:** 2020-02-25

**Authors:** Jasmina Vidic, Carole Chaix, Marisa Manzano, Marc Heyndrickx

**Affiliations:** 1 INRAE, AgroParisTech, Micalis Institute, Université Paris-Saclay, 78350 Jouy-en-Josas, France; 2 Institut des Sciences Analytiques, UMR 5280 CNRS, Université de Lyon, Université Claude Bernard Lyon 1, F-69100 Villeurbanne, France; carole.chaix-bauvais@univ-lyon1.fr; 3 Dipartimento di Scienze AgroAlimentari, Ambientali e Animali, via Sondrio 2/A, 33100 Udine, Italy; marisa.manzano@uniud.it; 4 Flanders Research Institute for Agriculture, Fisheries and Food (ILVO), Brusselsesteenweg 370, B-9090 Melle, Belgium; marc.heyndrickx@ilvo.vlaanderen.be; 5 Faculty of Veterinary Medicine, Ghent University, B-9820 Merelbeke, Belgium

**Keywords:** *Bacillus cereus*, spores, detection, biosensors, milk

## Abstract

Milk is a source of essential nutrients for infants and adults, and its production has increased worldwide over the past years. Despite developments in the dairy industry, premature spoilage of milk due to the contamination by *Bacillus cereus* continues to be a problem and causes considerable economic losses. *B. cereus* is ubiquitously present in nature and can contaminate milk through a variety of means from the farm to the processing plant, during transport or distribution. There is a need to detect and quantify spores directly in food samples, because *B. cereus* might be present in food only in the sporulated form. Traditional microbiological detection methods used in dairy industries to detect spores show limits of time (they are time consuming), efficiency and sensitivity. The low level of *B. cereus* spores in milk implies that highly sensitive detection methods should be applied for dairy products screening for spore contamination. This review describes the advantages and disadvantages of classical microbiological methods used to detect *B. cereus* spores in milk and milk products, related to novel methods based on molecular biology, biosensors and nanotechnology.

## 1. Introduction

The dairy industry has a long tradition of safeguarding the safety and quality of consumer milk, based on two main processes: cooling of the raw milk to temperatures below 7–10 °C until processing and heating the milk in a dairy plant. Production of different milk products demands various heating processes to be applied (pasteurization, ultra-high temperature (UHT) treatment, drying). The shelf life of pasteurized milk is mainly determined by the presence and growth of Gram-positive, rod-shaped aerobic endospore formers, of which members of the *Bacillus cereus* group are the most important. Thermophilic endospores resist the pasteurization process and may revive by germination and outgrowth and produce spoilage enzymes (proteases, lipases and phospholipases) in the pasteurized milk leading to off-flavors [1,2]. In addition, *B. cereus* is a well-known foodborne pathogen. Because of these risks, the dairy industry must constantly optimize and improve the processes that result in products that meet business and consumer demands and which can be exported over long distances and sometimes in unfavorable storage conditions without loss of quality. Despite further developments in the dairy industry in the last century, premature spoilage of milk continues to be a problem and causes considerable environmental and economic losses that are linked to the direct costs of recalls of products and indirectly linked by the image damage to the companies concerned. If the recall concerns a product containing milk powder, the recall costs may be greater than for consumer milk [3].

*Bacillus cereus* is a Gram-positive, rod-shaped, motile, spore-forming opportunistic pathogen that is commonly found in soil, air, grains, rice (row and cooked), vegetables, meat and milk due to the bacterial capability to grow at temperatures from 4 °C to 50 °C and resist heat and chemicals [4]. Some species from the *Bacillus cereus* group, also known as *Bacillus cereus sensu lato (s.l.)*, cause foodborne outbreaks in humans [5]. The group, as reported by Liu et al. [6], comprised until recently of nine officially closely related species: *B. anthracis*, *B. cereus sensu stricto (s.s.)*, *B. thuringiensis*, *B. mycoides*, *B. pseudomycoides*, *B. weihenstephanensis*, *B. cytotoxicus*, *B. toyonensis* and *B. wiedmannii*. From 2017 onwards, the *B. cereus* group has been expanded with another nine new species, which were all isolated from marine sediments: *B. paranthracis*, *B. pacificus*, *B. tropicus*, *B. albus*, *B. mobilis*, *B. luti*, *B. proteolyticus*, *B. nitratireducens* and *B. paramycoides* [7]. Three new species have been described based on a whole genomic sequencing comparison and could potentially also belong to the *B. cereus* group, but they have not (yet) been validly described according to bacterial nomenclature: *B. gaemokensis*, *B. manliponensis* (DSMZ, L.I. German Collection of Microorganisms and Cell Cultures GmbH, Inhoffenstraβe 7B, 38124, Braunschweig, Germany) and *B. bingmayongensis* (TEDA. School of Biological Sciences and Biotechnology Nankai University, Tianjin, China). In routine practice, it is difficult to distinguish between several of the different species of the *B. cereus* group, and therefore exact speciation is not often done, but rather a general allocation is given to the species group.

*B. cereus s.s.* causes mild to serious food poisoning, mainly through secretion of enterotoxin, causing diarrhea and emetic toxins [8], but also through some still non-elucidated mechanisms [9]. It was also found to be the etiologic agent of systemic and local infections in immunologically compromised and vulnerable individuals [5]. In same rare cases, *B. cereus* causes toxic shock syndrome in the central nervous system [5,10,11,12]. *B. anthracis* is responsible for causing a lethal disease anthrax in humans and animals.

*B. thuringiensis* is considered safe for humans, which enables commercialization and use of specific marketed strains as a bio-pesticide for pest controls as it causes lethal infections in insects with toxins, called Cry proteins [13]. *B. thuringiensis* has been reported in some cases of foodborne outbreaks, but it developed because classical routine detection methods did not allow to distinguish between *B. cereus* and *B. thuringiensis* [14,15]. The Cry genes encoded for the toxin show a high level of polymorphisms, which makes the differentiation of *B. thuringiensis* from other *B. cereus* strains difficult to define. However, the contribution of *B. thuringiensis* to foodborne outbreaks is still controversial but may be underestimated. Indeed, some *B. thuringiensis* strains used as biopesticide can produce modest levels of enterotoxins [14].

The psychrotolerant species *B. weihenstephanensis* and *B. mycoides* were, until recently, not considered foodborne pathogens. However, it has been demonstrated that *B. weihenstephanensis* can also produce emetic toxin [16]. It was also reported that some *B. pseudomycoides* isolates, which are considered as harmless microorganisms, may be potentially cytotoxic and possibly pathogenic [17,18]. *B. toyonensis* is used as probiotic and feed additive. In a recent study on *B. cereus* group isolates from spices, it was demonstrated that besides *B. thuringiensis*, some *B. weihenstephanensis* and *B. toyonensis*-like isolates can potentially produce enterotoxins [19]. Furthermore, *B. pseudomycoides* isolates were found to be potentially cytotoxic [18]. *B. cytotoxicus* associated with specific food products is the only thermotolerant member of the *B. cereus* group (it grows between 20 °C and 50 °C). It was shown to produce the highly toxic CytK1 variant, which was responsible for a deadly outbreak [20]. For the other most recent members of the *B. cereus* group, nothing is known about their pathogenic potential.

Consuming food contaminated by *B. cereus* may lead to gastrointestinal diseases, characterized by abdominal pain and non-bloody diarrhea that occur 4–16 h after eating. These symptoms are caused by the bacterial production of diarrheal toxins hemolysin BL, Hbl [21], nonhemolytic enterotoxin Nhe [21] and cytotoxin CytK [22] in the human small intestine. All three enterotoxins are cytotoxic and cell membrane pore-forming toxins [23,24]. Emetic disease may occur within 0.5–5 h after eating contaminated food as an acute attack of nausea, vomiting and profuse abdominal cramping due to a thermo- and acidic-stable non-ribosomal peptide, cereulide [25,26]. The mechanism of action of cereulide is not well documented but seems to be receptor-mediated [27,28]. In contrast to less heat-stable Hbl, Nhe and CytK toxins, which are produced in the small intestine and cause diarrhea as a result of a toxico-infection, cereulide can be present in contaminated foods and cause intoxication as it is resistant to heat, acid and proteolysis [29,30]. Specific peptides of the polypeptide toxins Hbl and Nhe can be easily detected through commercial immunological kits [31], while HPLC (High-Performance Liquid Chromatography) or mass spectroscopy are need for the other toxins [32,33,34].

*B. cereus* may survive under non-favorable conditions, such as under starvation or in a reduced oxygen or dry environment, for many years in the form of spores. Spores easily spread and can resist ow and high temperatures, desiccation, disinfectant agents, ionization, radiation and ultraviolet light. *B. cereus* spores are found in all categories of food (such as meat products, dairy products, vegetables, and rice) as they are widely distributed in soil, air, water, plants and animals. Milk and dairy products are particularly at risk because spores survive in pasteurized and other heat-treated milk products, such as milk powder and cheese. Fortunately, ultrahigh heat temperature (UHT) treatment, which is commonly used to produce consumer milk effectively, kills *B. cereus* spores [35]. Reconstituted infant foods are considered to be a high risk due to infant susceptibility to enteric bacterial pathogens because of their underdeveloped immune and metabolic systems. Powered infant formula, commercialized as a breast milk substitute, and powered follow-up formula, commercialized as an addition to breast milk, cannot be sterilized using current technologies because of the complex processing [15,36]. In this review, we summarize the contamination, prevention and detection of spores of *B. cereus* in milk and dairy products. We particularly focus on new analytical strategies to sense spore-contaminated milk.

## 2. Origin of Milk Contamination

*B. cereus* is widely distributed in nature and can contaminate foods primarily through soil and air [37,38]. Soil contains 50–380,000 CFU (colony-forming unit)/g and air <100 CFU/m^3^ of *B. cereus* spores [39]. The composition of the *Bacillus* microbiota in raw milk exhibits seasonal differences, with higher *B. cereus* spore counts in summer [39]. This is largely attributed to soil as a major source of *B. cereus* spores, to which cows are more exposed while pasturing during summer. During winter months, feed and bedding are considered the main contamination sources, as the cows are housed indoor in that period. These general observations were confirmed in a study conducted in Belgium, in which organic dairy farms were compared with conventional farms. Members of the *B. cereus* group were isolated more frequently in the late summer/autumn period and were also more frequently obtained in milk from organic dairy farms. This could be linked to more general outdoor grazing of cows on the organic farms compared to the conventional farms [40]. Depending on the country studied, seasonal changes in the composition of the *B. cereus* group population have been or have not been observed in raw milk, e.g., in Sweden higher psychrotrophic *B. cereus* levels were found in summer compared to winter [41], while in Japan, such a seasonal variation in psychrotrophic spore levels was not found [42].

The prevalence rate of *B. cereus* in raw milk is rather high, but usually at very low levels (<100 CFU/mL of raw milk) [39], although there probably exists farm differences depending on hygienic levels and housing systems of dairy farms. In the Netherlands, the average is 1.2 log spores of *B. cereus* per liter of raw bulk tank milk, and a maximum *B. cereus* spore limit in farm tank milk of 3 log spores per liter is required to achieve a shelf life for pasteurized milk of at least seven days [43]. Different types of cheeses in Turkey were positive for *B. cereus* at a rate of 10.4% and with counts up to 3.8 × 10^5^ CFU/g [44]. In fresh ricotta cheese, about 16% of the samples were positive for *B. cereus*, but at very low concentrations (<10 CFU/g) [45]. In recent studies on UHT-milk, *B. cereus* was found, which is unexpected in this type of product [46,47]. Possible explanations are an inadequate control of the UHT process or post-heat treatment contamination. Post-production, powders can be stored for extended periods and, in the absence of water, bacterial metabolic activity and growth is limited. Thus, drying milk prevents spoilage and product defects. However, under these conditions, bacterial spores can remain dormant until more favorable conditions are encountered, upon which germination and outgrowth can proceed [48,49].

Because of *B. cereus* ubiquitous presence in soil, water, and food processing environments, food contamination seems to be inevitable [50]. Although *B. cereus* spores have been detected in almost all food, they are mainly contaminants of raw foods, such as fresh vegetables and fruits, seeds, and raw milk. Spores of *B. cereus* can contaminate raw milk if hygienic milking conditions are not fully respected, but also via contaminated milking equipment or during transport from the farm to the dairy plants.

Contamination of milk powder for infants is one of the problems to be solved. It is interesting to note that the spore-forming bacterial flora present in the raw milk is different from the bacterial spore-forming flora present in the milk powders, in consequence of the industrial process used [51].

## 3. Legislation

*B. cereus* was among the primary microbes associated with baby food contamination, as reported by FAO/WHO (Food and Agriculture Organization of the United Nations/World Health Organization), and the third agent responsible for collective foodborne infections in Europe [38]. In 2005, EFSA (European Food Safety Authority) estimated that *B. cereus* accounts for 1.3% of all bacterial foodborne diseases [38]. In 2016, the European Union reported that 5.5% of outbreaks caused by a foodborne pathogen are attributed to *Bacillus* toxins as a causal agent [15]. Some dehydrated foods, including dried infant formulae and dried dietary foods, are consumed by potentially fragile consumers; therefore, the numbers of *Bacillus cereus* spores in these products should be as low as possible during processing, and good practices should be designed to reduce delay between preparation and consumption. Dried infant formulae and dried dietary foods for special medical purposes intended for infants below six months of age are the only food products for which there are official criteria in the EU for *B. cereus*: The target limit is 50 CFU/g with a tolerance limit of 500 CFU/g in a sampling plan comprising five sample units, of which one sample unit may be between both limits [52].

Foodborne diseases due to *B. cereus* are usually related to the presence of high numbers of cells or spores/g in the range of 10^5^–10^8^ in ingested food, although the amount regarded as hazardous can decrease to 10^3^–10^4^ cells or spores/g [38]. In several countries like Belgium, action limits (>10^5^ CFU/g or CFU/mL) are in place based on the EFSA recommendations. Spores of *B. cereus* are usually present in low numbers in raw milk (< 1 and up to 10–100 spores/mL), while the finished products, such as pasteurized milk, milk powder or cheeses, can contain about 10^2^–10^3^ spores/g during shelf life [43]. As explained above, the content of *B. cereus* in milk and dairy products may increase due to milk contamination with bacteria from soil, air or water and through storage abuse conditions.

## 4. Spores of *B. cereus*

To resist to environmental stress, an adaptive strategy of *Bacillus* cells is to transform into spores, which are called endospores because they are produced within a mother cell [53]. In the sporulation form, bacterium may stay dormant without nutrients for an undefined period but will germinate and again become a vegetative cell when conditions change to favorable. It is interesting to note that some food treatments, such as a sub-lethal heat exposure (at 65 to 80 °C), can act as triggers for germination [54]. *B. cereus* spores have great ability to survive at conditions like an unfavorable pH (from 1 to 5.2), a high temperature shock (such as 95 °C, for 2 min), or under antibiotic treatments (such as ampicillin, cephalothin and oxacillin), which, in contrast, their vegetative form cannot survive [4,31].

**Figure 1:**
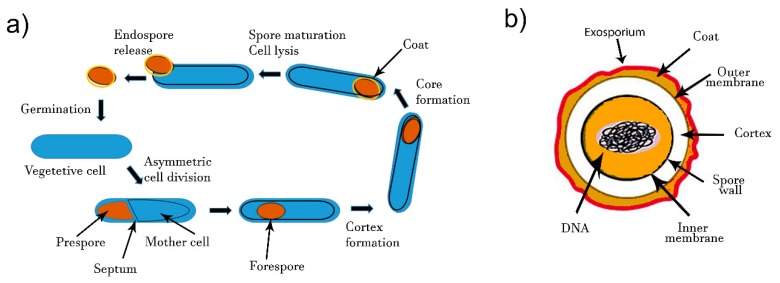
(**a**) Formation of spores by endospore-forming *B. cereus*. Upon unfavorable environmental conditions, the vegetative cell differentiates and enables spore morphogenesis. Mature spores are released and will germinated to give rise to a vegetative cell under favorable conditions. (**b**) Schematic diagram of bacterial spore structure.

The process of bacterial differentiation from cell to spore starts upon phosphorylation of the key transcriptional regulator, Spo0A [55]. For that, the starvation signals induce auto-phosphorylation of Spo0F kinase, followed by the phosphorylation of Spo0B kinase and Spo0A [56]. Phosphorylated Spo0A modifies expression of more than 500 genes [57], among which are some other regulators. About 100 genes are essential for the sporulation process [58,59,60,61,62]. The mineral composition of media may have a significant effect on sporulation. Manganese is especially an essential element for sporulation in the genus *Bacillus* [63]. The formation of endospores starts by an asymmetric cell division and DNA segregation (Figure 1a). Segregated DNA becomes separated from the rest of the cell by a double layer septum that is formed around it. This structure is called forespore (Figure 1a). Calcium dipicolinate (2, 6-pyridinedicarboxylic acid) stabilizes forespore formation and structure. Next, the peptidoglycan cortex is synthetized between the two layers and a spore coat is formed at the outside of the forespore. Finally, maturation of endospore and mother cell lysis enables spore release. The whole process takes around 8 h. Endospores contain several tick layers (Figure 1b). The endospores are resistant to higher temperature mainly due to their electronegative peptidoglycan cortex layer. The inner peptidoglycan layer surrounding the core enables endospore resistance to UV light and various bactericide chemicals. The inner membrane is surrounded by the germ cell wall, which becomes the membrane of the vegetative cell after germination [64]. Spores also contain some proteins, such as proteases, lyases, endonucleases and heat shock proteins that also contribute to the resistance of spores in unfavorable conditions [65].

Resistance of Bacillus cereus spores to external conditions indicates that their detection by an internal biomarker that should be extracted would be difficult. The more adapted analytical methods for spores detection in dairy products would be based on detection of spore’ surface epitopes or a surface structure. Spore preparation in controled laboratory conditions have shown that physicochemical propertis depend on sporulation medium and condition [66].

Various methods are used to induce *B. cereus* sporulation in the laboratory. For this, the bacterium is allowed to multiply to the wanted growth level (usually to mid-exponential phase), and then cells are exposed to a stress that induces their transformation. For instance, *B. cereus* cells can be transferred by plating from a rich broth, such as brain-heart infusion (BHI) that support bacterial proliferation, to a nutrition agar complemented with 0.05 g/L MnSO_4_. To avoid starvation in the presence of Mn^2+^-ions, 90–95% of *B. cereus* cells sporulate within few days incubation at 30 °C or 37 °C [67]. Spores formed by various species from the *B. cereus* group have a similar size (about 1 µm) and morphology. However, the structure of the superficial layer, named the exosporium, can be modified to some extent by growing the medium or sporulation condition. Overall, findings obtained in laboratory conditions indicate the high risk associated with the presence of *B. cereus* spores in thermally processed and packaged milk products that may have high salt concentration, absence of oxygen, or are conserved at refrigerated temperatures.

## 5. Detection Methods

### 5.1. Classical Methods

**Figure 2:**
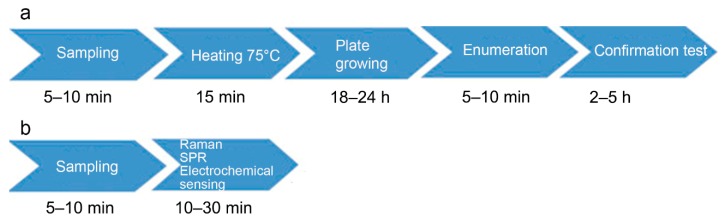
Envisioned spore diagnostic workflow by a classical method (**a**) and biosensors (**b**).

Conventional detection of *B. cereus* spores or cells involves plate count method analysis and toxin detection by commercial immunological kits or a combination of liquid chromatography and mass spectrometry (Figure 2). In addition, PCR-based methods are frequently used to evidence the toxin genes. Official methods to enumerate and detect the *B. cereus* group are ISO 7932 (for direct plate counting) and ISO 21,871 (for detection and counting of low numbers through the most probable number). Both methods give no indication of the ability of the bacteria to produce toxins, nor do they differentiate *B. cereus* sensu stricto from other bacteria of the *B. cereus* group; therefore, the result is presented as “presumptive *Bacillus cereus*”. Genetic molecular methods detect the presence of certain genes, so enable discrimination of *B. cereus* from other bacilli. Methods for detection of toxins and strain characterization for the production of toxins are not standardized. As a result, no official method is available for routine use to screen food. However, some advanced methods are under investigation [34,68].

The traditional microbiological methods for general detection of spores include pour-plating on plate count agar and microscopic observation. *B. anthracis* and *B. thuringiensis*, which are subtyped in serotypes, are also detected by strain iso-typing using specific antibodies. As for the detection of spores of other aerobic spore-forming bacteria, the standard plating procedure to specifically detect spores of *B. cereus* considers the thermal treatment of the homogenized sample solution at 75 °C for 15 min before plating 1 mL of the solution on the specific medium MYP ((Mannitol Egg Yolk Polymyxin Agar) and confirmation of suspected colonies by a hemolysis test [68]. This thermal treatment kills the vegetative forms, allowing the spore, a resistant form, to grow on nutrients. By plating serial dilutions of the treated homogenate, it is possible to evaluate the spore concentration. In that way, the method quantifies the survival of *B. cereus* spores after heat treatment of food products.

Official plating methods of general spore or specific *B. cereus* detection or enumeration described above require at least two-day incubation to provide results. However, endospores can be detected in more rapid manners (Figure 2). One of the rapid methods that has been used for many years to quantify spores in a bacterial culture is based on the utilization of a hemocytometer. For instance, malachite green at 0.5% water solution colors spores and allows their visualization under simple optic microscopy. This method, proposed by Kiozuka and Tochikubo in 1991, was useful for dormant spore detection [69]. However, there is no evidence that it can be applied for food analysis.

### 5.2. PCR and LAMP

Traditional techniques can lead to misidentification as there is a high phenotypic similarity among the *B. cereus* group species in terms of pathogenicity, morphology of colonies, mobility and growth kinetics or sporulation efficiency. Molecular techniques are an alternative to classical methods, although it is difficult to extract DNA from spores that are highly resistant against chemical or enzymatic lysis. Nevertheless, various methods can be used to release DNA, e.g., sonication or mechanical lysis with glass beads. Some commercial kits for spore disruption are also available [70]. However, in many diagnostic protocols, spores are allowed to germinate and DNA is extracted from lysed vegetative cells. Martinez–Blanch et al. described a quantitative real-time PCR to evaluate spore concentration, using as a target the pc-plc gene encoding for phosphatidylcholine-specific phospholipase involved in the hemolysis activity [71]. The protocol allowed the detection limit for *B. cereus* in artificially contaminated reconstituted infant formula of about 4 spores per reaction or 60 spores per mL, using the curves obtained with an efficiency of 1.09. In another study, the Ruggedized Advanced Pathogen Identification Device (RAPID) was employed to detect spores of Bacillus anthracis in raw milk, with the limit of detection (LoD) down to 2500 spores/mL [72]. The protocol consisted of the utilization of a commercially available DNA extraction kit, portable equipment for real-time PCR and commercially available primers and probes, developed to detect either the protective antigen gene or the lethal factor gene with the RAPID [72].

Since milk typically contains a too low concentration of spores to be directly tested by a PCR method, a time-consuming microbiological enrichment step is needed prior to detection in routine analysis. In addition, milk contains a high concentration of ions and fats that have been shown to inhibit real-time PCR. To overcome problems of low spore and high ion and fat concentrations, Fisher et al. proposed an aptamer-based trapping protocol for milk analysis [73]. Magnetic capture is an excellent strategy for extracting and concentrating bacteria or spores from complex food matrices and/or highly diluted solutions [74]. The spore trapping process was based on the use of aptamer-functionalized silica magnetic beads for the specific capture and concentration before detection by specific real-time PCR. The aptamers were designed to specifically detect *B. cereus* spores with high affinity (K_D_ in the nM range). Such aptamer-decorated magnetic beads successfully trapped spores from milk with different fat contents, showing enrichment factors up to six-fold. This strategy improved the limits of detection of the subsequent PCR test. Indeed, after several washing steps with pure water, trapped spores were analyzed by a highly specific real-time PCR assay to gain a detection limit of 10^3^ CFU/mL for *B. cereus* in milk. However, this is still not a very sensitive detection limit, since it is not sufficient for raw milk where the contamination level is still much lower. Other molecules, such as the bacteriophage cell wall-binding domain, can be used to pre-concentrate *B. cereus* from complex food matrices [75]. Some rapid tests for detection of *B. anthracis* spores based on nucleic acid detection showed a very low limit of detection (LoD) of 1 to 30 spores per reaction [76,77,78]. However, to reach such a low limit of detection, a clean and concentrated starting material is needed, which can hardly be the case with contaminated milk and dairy products.

An alternative nucleic acid amplification technology is loop-mediated isothermal amplification (LAMP), which works at an isothermal temperature, which is advantageous for rapid and point-of-care portable detection systems [34]. A rotate and react SlipChip (RnR-SlipChip) was developed for simultaneous visual detection of multiple bacterial pathogens by LAMP, including *B. cereus* [79]. After sample loading, one-step rotation allowed immediate mixing and reaction of target bacteria with the reagents on the chip, allowing visual identification in 60 min with a success rate of 100%. It has to be further investigated if this technology can be adapted to also detect spores of *B. cereus*.

### 5.3. Dipicolinic Acid Detection

The spore-specific dipicolinic acid, present as a Ca^2+^ chelate complex, is a unique and characteristic biomarker for endospores of *B. cereus*, but also for other bacterial spores produced by other aerobic spore-forming species and Clostridium, but not in spores formed by other organisms, such as molds. Dipicolinic acid makes 5% to 15% of the total dry mass of the spores and may be released during heating or hydrolysis [80]. Dipicolinic acid detection provides good estimation of bacterial spore content in suspicious samples and could be used for a rapid screening of milk and dairy products. In the literature, different techniques have been described for dipicolinic acid detection. Several optical methods, such as spectrophotometry, Raman/surface-enhanced Raman scattering (SERS) and infrared spectroscopies, fluorescence and photoluminescence, have been applied to dipicolinic acid detection. Rosen proposed to color bacterial endospores by a suspension of terbium chloride, which reacts with the calcium dipicolinate [81]. Formed terbium-(III)-dipicolinate anion produces photoluminescence that can be easily detected. The LoD obtained using 31.4 µM TbCl_3_ b was 4.4 × 10^5^ CFU/mL. Gültekin et al. [82] described the elaboration of molecular imprinted polymer (MIP)-nanoshells on gold-silver nanoclusters. These MIP-nanosensors were developed with a specific cavity for dipicolinic acid interaction. The binding affinity was investigated by fluorescence and K_D_ was calculated between 5 × 10^−8^ to 1 × 10^−7^ M. This strategy allowed *B. cereus* spore detection via dipicolinic acid quantification in a concentration of 10^4^ CFU/mL. Baig and Chen have used glutathione-capped gold nanoparticles complexed with Ca^2+−^ions (Ca^2+^-AuNP@GSH) through GSH-Ca^2+^ chelation as a sensing agent for dipicolinic acid from complex matrices [83]. In the presence of dipicolinic, Ca^2+^-AuNP@GSH is dissociated to the Ca^2+^-dipicolinic complex and AuNP@GSH, which desegregates nanoparticles and induces a color change of the solution. The feasibility of using this sensing method for quantitative detection of dipicolinic acid was demonstrated in lysate samples prepared from the *B. cereus* spore suspension.

Raman spectroscopy is particularly adapted for dipicolinic acid detection. In SERS, a single spore (or in bulk) in a spatially confined zone of enhanced electromagnetic field generates a highly specific spectral signature within seconds. SERS has many advantages, such as sensitivity, selectivity, no need for biomarker extraction, and no interference from aqueous media that makes it attractive for biosensing applications. Chan et al. described an analytical technique combining optical trapping and confocal Raman spectroscopy to detect and identify a single spore particle. *B. cereus* spores were identified based on their intrinsic Raman signature, which revealed to be specific [84]. Raman spectroscopy coupled with SERS-active gold nanoparticles was used to detect and discriminate among five Bacillus spores by using the SERS method [85]. In a similar way, peptide-functionalized silver particles embedded in a porous silica sol-gel was used for selective capture and detection of *B. cereus* spores via dipicolinic acid signature by SERS [86]. In 2013, Cowcher et al. described a SERS portable device for quantitative detection of bacterial spores. The technique was also based on dipicolinic acid detection and quantification and proved specific to *B. cereus* and *B. subtilis* spores. An approximated LoD of 1100 spores in a 200 µL sample volume was reached by the technique [87]. These performances are close to those of real-time PCR, as previously described.

Spore content in Bacillus species’ samples was also determined by mass spectrometry, as described by Beverly et al. [88]. The detection of dipicolinic ions at *m/z* 167 was used to quantify various degrees of sporulation directly in live bacteria samples with good accuracy. Nevertheless, detection methods based on Raman spectroscopy or mass spectroscopy require expensive and sophisticated instrumentations.

The methods for detecting spores by quantifying dipicolinic acid are rarely applied in complex matrices, such as the milk or dairy industry. We should also mention the work of Han et al. [89], who developed a rapid enumeration of spores in raw milk through analyzing dipicolinic acid. In this assay, the concentration of dipicolinic acid in raw milk was obtained by measuring the solution absorbency and converting it into endospore numbers using endospore-forming pure cultures as standards. The LoD was 1.46 × 10^3^ CFU/mL.

### 5.4. Colorimetric Detection (Lateral Flow)

Quinlan and Foegeding used monoclonal antibodies for spore isotyping [90]. Antibody B183 and B48 reacted very strongly with *B. cereus* spores, but a light reactivity was also detected for *B. subtilis* subsp. globigii and *B. megaterium* spores, indicating cross reaction. Using immunogold staining they showed that both antibodies were bound to surface epitopes of spores. Such antibodies enable detection of whole spores in rapid tests, such as lateral flow immunoassays.

Lateral flow immunoassays attract growing interest for the development of user-friendly rapid tests to monitor food safety. The first principal advantage of lateral flow tests is that they can provide a colorimetric signal that can be assessed by the naked eye without any instrumentation. The second advantage lies in the movement of the sample through the paper carrier of the test, allowing separation of molecules from the sample before detection. This is particularly important regarding the complexity of food matrices and usually requires complicated sample preparation prior to analysis. Wang et al. coupled a super-paramagnetic separation with a lateral flow test to detect B. anthracis spores in water and milk [91]. To adapt the lateral flow test for the detection of large-sized objects, spores were firstly captured by paramagnetic beads, decorated with a specific antibody and then the formed immunocomplexes were deposited on the sample pad of the lateral flow test. Each spore was bound to numerous beads because they presented many surface epitopes. In consequence, immunocomplexes aggregated into large particles that could not flow through the paper stip. Instead, they were retained near the sample pad of the strip and generated a retention line. Super-paramagnetic beads mixed with spore free milk samples migrated over the paper strip, and no retention line was formed.

**Figure 3:**
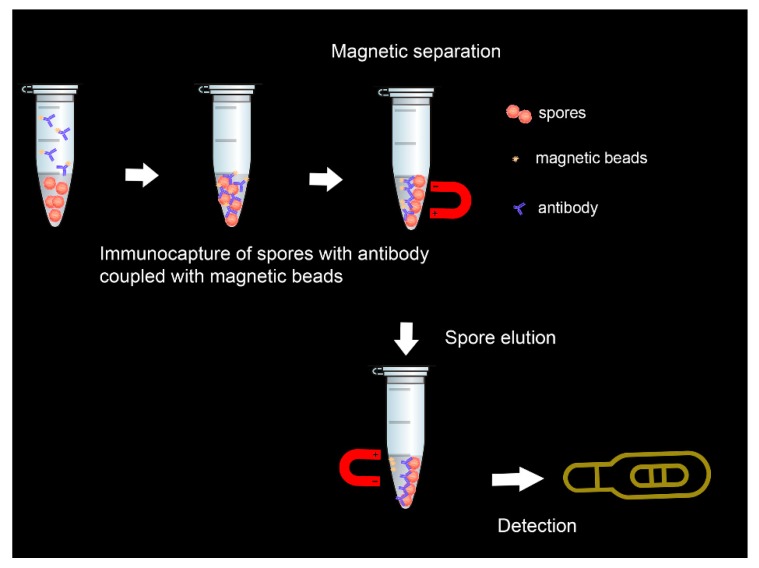
Illustration of the immunomagnetic lateral flow sensing principle.

In another study that combined magnetic separation of spores from water and milk samples with the lateral flow detection, spores were eluted from functionalized paramagnetic beads before detection [92]. Anti-spore antibodies coupled with carboxyl coated magnetic beads captured spores from milk samples within 30 min (Figure 3). The resulting complex was dissociated with formamide-EDTA (2,2′,2″,2‴-(Ethane-1,2-diyldinitrilo)tetraacetic acid ) solution for 30 s in a microwave oven, and then eluted spores were evidenced by the lateral flow test within 10 min.

Lateral flow assays were developed using different antibodies that recognized high affinity spores of *B. anthracis*. Both lateral flow set-ups can potentially be extended to spores of other *Bacillus cereus* species in milk samples. Some of the available antibodies are highly specific, while others can be used for *B. cereus* sensu lato spore detection. Alternatively, new antibodies should be raised against a soluble exosporium fraction of targeted *B. cereus* to enable detection of its specific whole spores.

### 5.5. Biosensors

**Figure 4:**
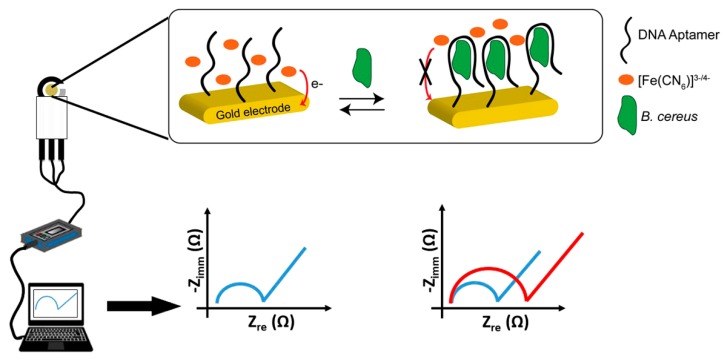
Illustration of principle for *B. cereus* spore detection and electrochemical set-up for signal measurement using a miniaturized aptasensor (from Reference [97]).

Due to the low number of recognition elements (such as antibodies, aptamers) that enable efficient and specific targeting of whole bacterial spores, only a few biosensors have been developed in the literature. Biosensors attract considerable attention because of their simplicity, sensitivity and rapidity (Figure 2). Electrochemical biosensors are the most rapidly growing compared to other methods, because they are low cost, sensitive and easy to miniaturize [93,94,95]. In 2012, Bruno and Carrillo reported an aptameric sequence, which specifically binds to spores of *B. anthracis* and *B. cereus* [96]. Based on the binding properties of this sequence, a label-free impedimetric aptasensor has been developed recently (Figure 4) [97]. *B. cereus* spores were detected with a linear range between 10^4^ CFU/mL and 5 × 10^6^ CFU/mL, with a detection limit of 3 × 10^3^ CFU/mL. The selectivity of the sensor proved efficient. The sensor also detected *B. subtilis* spores but with a slightly lower signal than for *B. cereus*, while no response was observed in the case of *Legionella pneumophila* and *Salmonella typhimurium* bacteria.

Surface plasmon resonance (SPR) is a sensitive method well suited to the label-free detection of microorganisms. Wang et al. used this technique for specific detection of *B. anthracis* spores through the specific recognition of a monoclonal antibody-functionalized sensor [98]. A detection limit as low as 10^4^ CFU/mL was reached in the 40 min analysis. In another study, *B. cereus* vegetative cells were detected by phage endolysin modified SPR chips with a limit of detection of 10^2^ CFU/mL by using a subtractive inhibition assay [99]. Such subtractive inhibitory assays may potentially increase the sensitivity of spore detection by a SPR sensor.

In view of the literature on spore detection methods, the development of new approaches appears necessary in order to develop more sensitive (LoD < 10^3^ CFU/mL) and specific analytical tools that can differentiate the spore species with good accuracy.

## 6. Conclusions and Perspectives

To ensure microbiological quality and consumer safety, strict controls and hygienic conditions are observed by the dairy industry, as recommended by guidelines of good dairy farming [100] and hygienic conditions at dairy plants (ISO 8086:2004). The control of environmental parameters (temperature, pH, water activity, salinity, atmosphere, presence of additives) can help the control of *B. cereus* proliferation in foods. The ability to produce spores make *B. cereus* capable to escape processing conditions carried out by the food industries to preserve products and to eliminate or reduce the bacterial number in the final product. Heat treatments commonly used by the food industry require longer treatments to efficiently eliminate *B. cereus* spores [101]. Moreover, depending on the heating conditions, pasteurization can either provoke activation of germination or growth of spores of *B. cereus*. This may result from the failure to recover or repair non-lethal injuries in this products at this temperature, as can be seen by the steady rate at which the spores died during storage at 7 °C [102]. Unfortunately, high heat procedures cannot be employed for all dairy products, especially not for infantile milk because it may destroy the nutritive quality of the milk. Subsequently, screening of milk and milk products for *B. cereus* spore contamination is of high importance for the dairy industry, which is looking for new cost-efficient, rapid and easy to use methods.

In this review, we have summarized the main problems that arise from the milk contamination by *Bacillus cereus* spores and remarkable advances in the development of novel sensitive assays for direct spore detection. Currently used plate-based methods for spore detection in milk and dairy products are time consuming, expensive, and need highly trained personnel. Inherent sensitivity, simplicity, and low cost of novel analytical methods, such as LAMP, electrochemical biosensors and lateral flow tests, start to attract more attention in the dairy industry. However, although novel portable assays display many advantages over currently used methods, they are not easily implementable, partially because only a few are available commercially. Nevertheless, it is evident that portable and cost-efficient biosensors for spore detection will be employed in the future to help milk producers, retailers, authorities and even consumers to increase microbiological quality in dairy products with minimal investment. Actual developments based on the combination of novel recognition elements, analytical technics and novel materials enabled significant improvements in microbiological analysis compared to current analytical systems. However, efforts should be made to take these assays from the laboratory bench to practical applications and mass production. The public and industrial demand for food safety will drive investment into biosensor design, marketing and commercialization.
